# Supplementary material for: Determinants of physical activity participation among U.S. adolescents aged 12–17: A study of key factors
Source: PLoS One. 2025 Jul 3;20(7):e0327536. doi: 10.1371/journal.pone.0327536 (PMC12225791; doi:10.1371/journal.pone.0327536)
Supplement: Attachment — Submitted filename: 1.docx [file pone.0327536.s001.docx]

1.The format in the paper needs adjustment. Line spacing, font size, paragraph spacing.

2.The section on data results in the paper could use more charts and graphs for illustration, such as family structure, education levels, etc.

3.The specific amount of data collected for this paper should be detailed at the end of the paper. It is unclear whether the data found online and the data used for analysis in this paper are consistent.

4.In the results analysis section, the source needs to be cited. For example, the content in cluster2 needs to be substantiated. How were the examples in the paper obtained? Were they extracted from online sources or analyzed from other papers?

5.Reference [19] draws conclusions based on a study focused on Brazil. It is unknown whether there is a positive correlation with the subjects of this paper. It is necessary to use American research literature for validation.

6.Could the recommendations section of the paper be expanded? The analysis yielded good results, but corresponding strategies are lacking.
